# BSI-MVS: multi-view stereo network with bidirectional semantic information

**DOI:** 10.1038/s41598-024-55612-6

**Published:** 2024-03-21

**Authors:** Ruiming Jia, Jun Yu, Zhenghui Hu, Fei Yuan

**Affiliations:** 1 School of Information Science and Technology, North China University of Technology, Beijing, 100144 China; 2 Hangzhou Innovation Institute, Beihang University, Hangzhou, 310051 China; 3 Institute of Information Engineering, Chinese Academy of Sciences, Beijing, 10085 China

**Keywords:** Multi-view stereo, Bidirectional-LSTM, 3D reconstruction, Transformer, Electrical and electronic engineering, Computer science, Information technology

## Abstract

The basic principle of multi-view stereo (MVS) is to perform 3D reconstruction by extracting depth information from multiple views. Most current SOTA MVS networks are based on Vision Transformer, which usually means expensive computational complexity. To reduce computational complexity and improve depth map accuracy, we propose a MVS network with Bidirectional Semantic Information (BSI-MVS). Firstly, we design a Multi-Level Spatial Pyramid module to generate multiple layers of feature map for extracting multi-scale information. Then we propose a 2D Bidirectional-LSTM module to capture bidirectional semantic information at different time steps in the horizontal and vertical directions, which contains abundant depth information. Finally, cost volumes are built based on various levels of feature maps to optimize the final depth map. We experiment on the DTU and BlendedMVS datasets. The result shows that our network, in terms of overall metrics, surpasses TransMVSNet, CasMVSNet, CVP-MVSNet, and AACVP-MVSNet respectively by 17.84%, 36.42%, 14.96%, and 4.86%, which also shows a noticeable performance enhancement in objective metrics and visualizations.

## Introduction

MVS technology facilitates a profound interaction between the digital and real worlds through 3D reconstruction. Traditional methods for 3D reconstruction^1,2^ based on geometric shapes can be categorized into various approaches depending on the inclusion of prior conditions. These approaches include contour-based methods, focused area methods, motion-based methods, and others. Traditional 3D reconstruction methods based on visual geometry primarily utilize the 3D geometric information present in 2D images as prior knowledge. This technique significantly restores the 3D scene without additional conditions. By leveraging the inherent geometric cues captured in the 2D images, these methods can deduce or derive the intrinsic 3D structure of the scene. However, traditional MVS methods, based on an ideal Lambertian scene and strict geometric relationships, may encounter challenges in reconstructing complex geometries or texture-free areas, leading to holes and texture blending issues.

With increasing computing power and the continuous advancement of deep learning, there has been a surge in research utilizing deep learning techniques for MVS tasks. MVS essentially uses prior geometric knowledge to recover spatial 3D shapes. Deep learning based methods do not discard this principle; instead, they employ neural networks to facilitate the process of geometric reconstruction. These methods learn the matching relationships between images from different viewpoints, enabling more robust 3D reconstruction. MVSNet^3^, as a learning-based method, introduces a breakthrough approach by transforming the 3D reconstruction issue into a deep map inference issue. This innovative methodology can be roughly divided into four key steps: image feature extraction, cost volume construction, cost volume regularization, and depth estimation^4^. By adopting this framework, MVSNet pioneers the use of deep learning techniques to achieve multi-view 3D reconstruction, paving the way for significant advancements in the field.

For the past few years, a new approach to 3D reconstruction incorporating the Transformer^5^ model, originally used for natural language processing, has emerged on the basis of deep learning. This approach employs the Transformer model for the feature extraction phase of 3D reconstruction, introducing a new perspective and potential improvements to the field. Thanks to the attention mechanism and the contextual aggregation of location encoding, the Transformer model has the ability to capture global information and semantic details of relevant locations. However, integrating the Transformer model into the 3D reconstruction field presents a significant challenge for the underlying hardware infrastructure. The Vision Transformer^6^ divides the input image into a series of patches and performs operations on them, resulting in a substantial increase in computational complexity. This can make it difficult to handle high-resolution images and slow down the convergence of the entire network.

Additionally, there is a limitation on the sequence length that the Vision Transformer can effectively handle. Longer sequences may result in information loss during the processing stage. This constraint needs to be considered when applying the Vision Transformer to 3D reconstruction tasks to ensure that important details and contextual information are adequately preserved in the reconstructed output.

The global attention mechanism in the Vision Transformer model can make the network more sensitive to noise in the input sequence during the 3D reconstruction process. This sensitivity to noise can potentially impact the quality and accuracy of the reconstructed output. Recently, there has been a resurgence in convolutional neural networks (CNNs) for sequence modeling. A notable approach involves utilizing BiLSTM^7^, like the Sequencer^8^ model, to address the task. Unlike the attention mechanism employed in Transformers, Sequencer utilizes BiLSTMs to process time sequences in width and height directions. Additionally, feature fusion is performed using convolutions, enabling better attention to temporal information. This alternative approach offers an effective solution for modeling sequences, considering both the spatial and temporal aspects, and has shown promising results in various applications.

We propose a MVS network with Bidirectional Semantic Information for 3D reconstruction called BSI-MVS. Our network utilizes a BiLSTM approach to spatially and temporally combine information for improved model generalization and enhanced 3D reconstruction accuracy. In BSI-MVS, we adopt a spatiotemporally combined approach incorporating a Multi-Level Spatial Pyramid module. This module enables the construction of a spatial pyramid at multiple scales, enabling the entire network to capture and encompass a diverse set of spatial semantic information. BSI-MVS can capture details and contextual information across different spatial resolutions by integrating information from multi-scales. This spatial pyramid construction enhances the network's ability to handle variations in spatial structures and improves the overall performance and accuracy of the MVS network. The coarse-resolution feature maps obtained from the Multi-Level Spatial Pyramid are individually processed in the horizontal and vertical directions using BiLSTM to address the problem of long-term dependency.

The following are the essential contributions of this paper:

- We introduce an MVS network with Bidirectional Semantic Information for reconstructing low-resolution images in the MVS task to solve the calculation accuracy problem.
- We propose a Multi-Level Spatial Pyramid module (MLSP) and a Bidirectional-LSTM module (BiLSTM) for the feature extraction stage. For low-resolution images, The MLSP can enhance the robustness of BSI-MVS by constructing multi-scale information. Then, using the BiLSTM module at each level of the feature pyramid allows for enhancing the understanding of contextual semantic information.
- We have benchmarked our network against pre-existing methods on a DTU dataset^9^ widely used by the MVS task. In the end, our proposed network achieved superior results. Furthermore, we evaluated the network's visualization capabilities on the BlendedMVS dataset^10^, which showed improved visualization results compared to other approaches.

## Related work

### Vision transformer-based MVS

Inspired by human visual perception, Vision Transformer mechanisms enable efficient image scanning to extract relevant information about the target of interest. As the Vision Transformer becomes more widely used in computer vision, the Vision Transformer is also used in 3D reconstruction for better feature extraction. TransMVSNet^11^ is the first network to use the Vision Transformer for the MVS task, which uses the Vision Transformer for global contextual perception within and between images. AACVP-MVSNet^12^ introduces an attention layer to improve feature extraction and uses a similarity metric to aggregate cost volume. Liao, Jinli, et al.^13^ proposed to use an improved window attention mechanism for the global feature aggregation and local feature matching phases of 3D reconstruction with the aim of reducing redundancy and increasing smoothness. SENet (Squeeze-and-Excitation Network) is a novel approach that leverages convolutional neural networks (CNNs) and attention mechanisms to model channel relationships. The attention mechanism in SENet is designed to learn channel dependencies in order to highlight valuable information within each channel while suppressing irrelevant or redundant features. By effectively capturing and recalibrating channel-wise feature responses, SENet enhances the network's ability to focus on informative features, leading to improved performance and better utilization of channel information within CNNs.

While the Vision Transformer-based MVSNet has shown improvements in the quality of 3D reconstruction, it is crucial to consider the following limitations. The global self-attentiveness mechanism employed in the Vision Transformer introduces challenges such as increased network parameters and computational complexity. This network can result in higher resource requirements and longer inference times. Moreover, the inherently global nature of self-attention may make the network more sensitive to environmental disturbances and variations, potentially impacting the overall stability of the reconstruction process. These trade-offs between improved reconstruction quality and increased computational burden need to be carefully considered when applying Vision Transformer-based approaches in the context of MVS.

### CNN-based MVS

MVSNet, as a CNN-based method^14^, introduced the concept of transforming the 3D reconstruction problem into a depth map inference problem. This novel approach paved the way for leveraging deep learning techniques in multi-view 3D reconstruction. Cas-MVSNet^15^ utilizes a cascading architecture with multiple sub-networks for 3D reconstruction. CVP-MVSNet^16^ is a system that utilizes a compact and lightweight network to construct a pyramid of cost volumes. This approach allows for achieving enhanced resolution in 3D reconstruction. By leveraging this technique, CVP-MVSNet can generate more detailed and accurate reconstructions. GeoMVSNet^17^ uses the geometric prior to guide the fusion process for better feature fusion. In the MVS task, it is common to apply cost volume regularization^18^ to smooth the features. However, this regularization technique alone cannot completely address the issue of ambiguous feature matching caused by reflections or texture-free regions with unreliable 2D image features. These challenges can still cause imprecision in the reconstructed 3D models. Hence, it is crucial to focus on learning influential and representative characteristics during the feature extraction stage to enhance the generalizability of MVS systems. By obtaining high-quality features, the MVS algorithm can better handle challenging scenarios, such as reflections^19^ and texture-free regions, leading to more reliable and accurate 3D reconstructions.

Experimental results show that a well-designed CNN can achieve results beyond the Vision Transformer. ConvNext^20^ is a novel architecture that builds upon the SwimTransformer^21^ model. It incorporates convolutional layers to achieve an attention-like mechanism, surpassing the performance of the original SwimTransformer model. By leveraging convolutional operations, ConvNext enhances the model's ability to capture relevant features and improve its overall performance in various tasks, while FLOPS are significantly lower than SwimTransformer. InceptionNext^22^ achieves superior performance using separable convolution compared to SwimTransformer with significant reductions in both the number of parameters and FLOPS. Sequencer utilizes LSTM instead of an attention mechanism for natural language processing. The network leverages bidirectional long-short-distance memory to perform classification tasks by serializing feature maps. And BH-RMVSNet^23^ uses bidirectional hybrid LSTM for the cost volume regularisation in MVSNet to improve memory efficiency. Hence, replacing the Vision Transformer for feature extraction in 3D reconstruction with convolutional neural networks can improve network performance while reducing the number of parameters.

## Method

**Figure 1:**
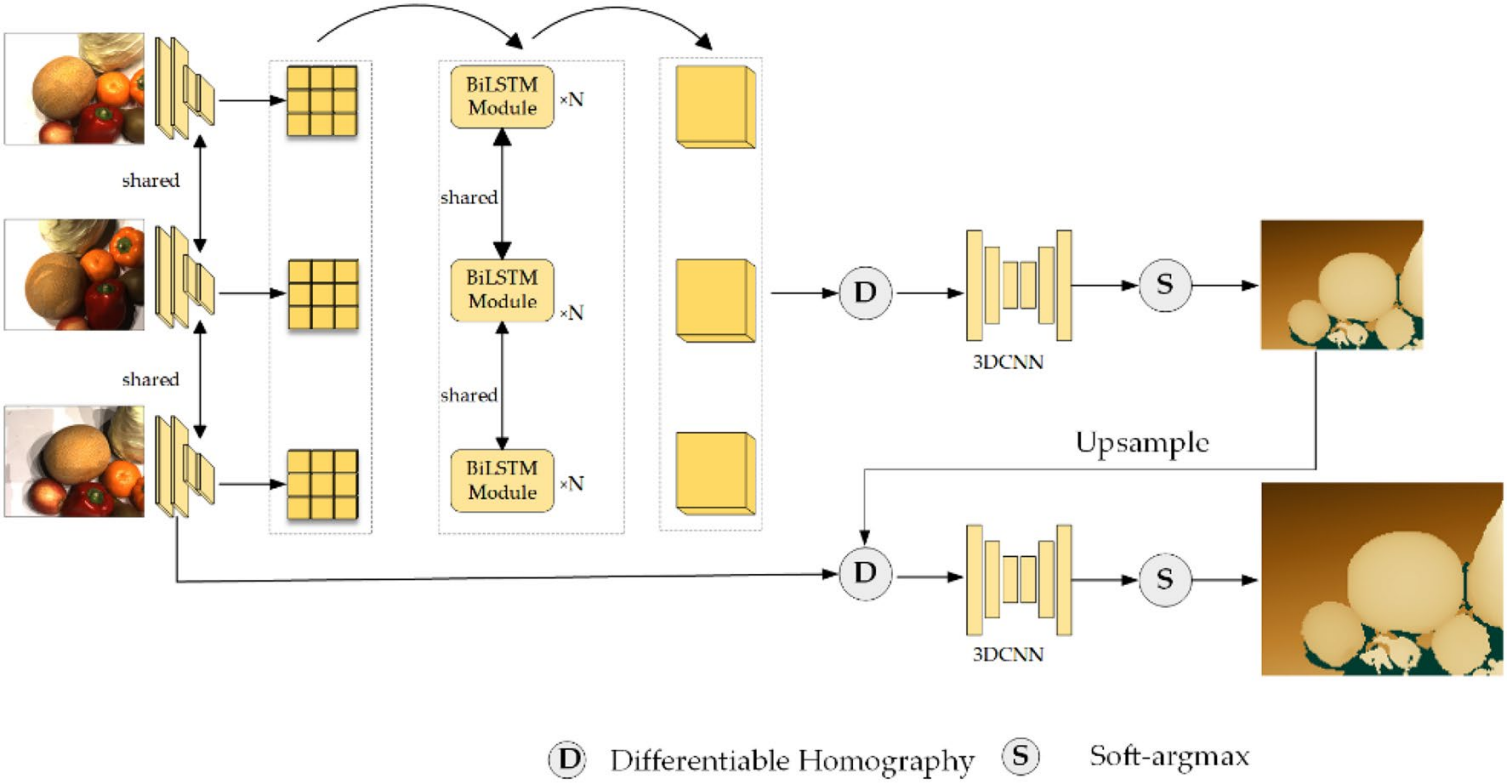
The network structure of BSI-MVS.

To optimize the utilization of visual data and enhance the fidelity of the reconstructed 3D models, we have proposed an innovative method called BSI-MVS. Figure 1 depicts the detailed process of our proposed method, which is divided into three main stages: spatiotemporal feature extraction, cost volume regularization, and depth estimation. The input to our network is a reference image $I_{0} \in {\mathbb{R}}^{H \times W}$, where H and W are the height and width of the image, N source images $\left\{ {I_{{{\text{i}} = 1}}^{N} } \right\}$, and the camera's internal and external reference matrices for the corresponding viewpoint $\left\{ {K_{i} ,R_{i} ,{\text{t}}_{i} } \right\}_{i = 0}^{N}$. In our proposed network, the initial stage involves passing all input images through a Multi-level Pyramid module with weight sharing. In the second step of our proposed network, the low-resolution feature maps obtained from the previous step are inputted into the BiLSTM module. By leveraging the capabilities of the BiLSTM module, the network can better understand and encode relevant contextual information in the feature maps. Finally, similar to standard MVS networks, our approach performs cost volume construction, regularization, and in-depth reasoning.

### Multi-level spatial pyramid (MLSP)

**Figure 2:**
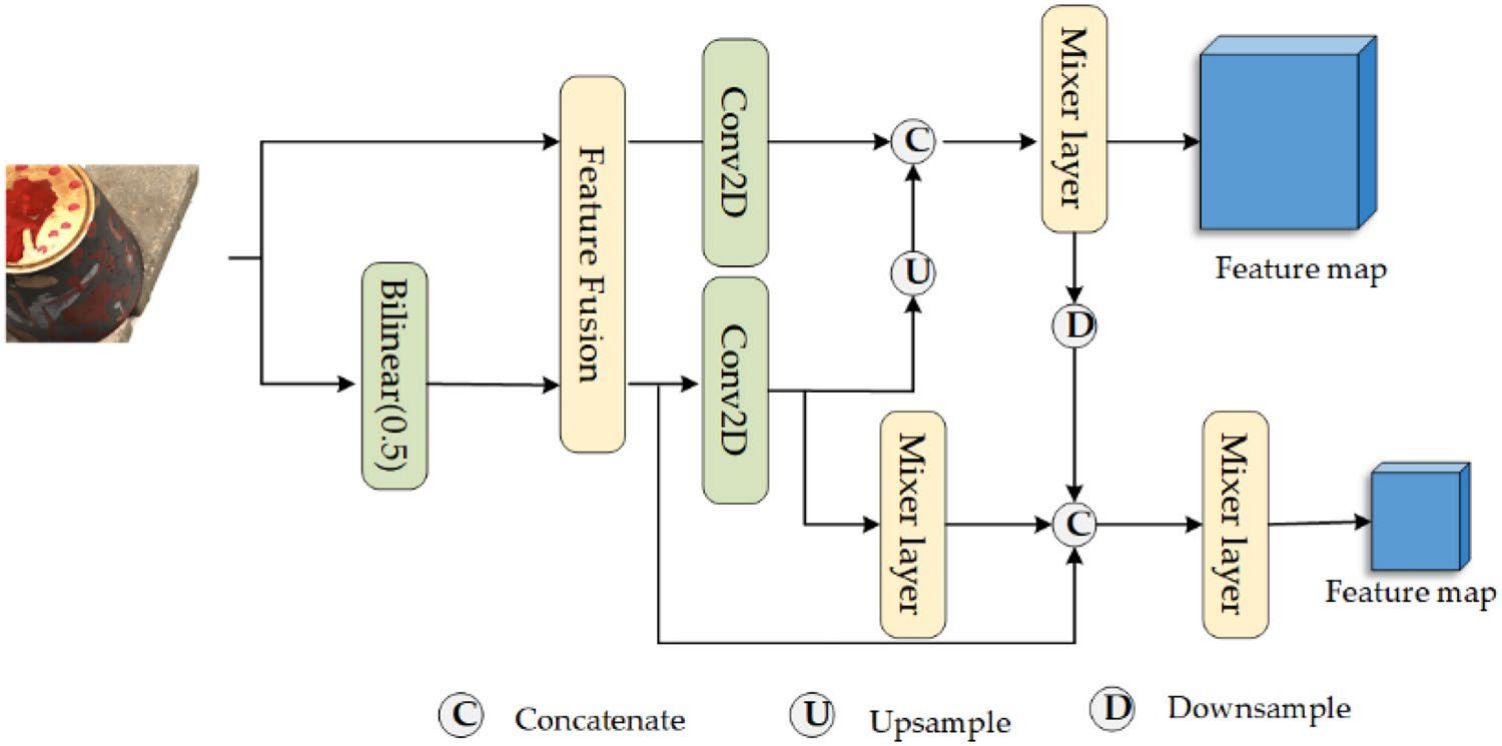
The structure of MLSP Module.

Our proposed MLSP module facilitates the interaction of data across diverse spatial levels, enabling the fusion of information at various resolutions. It achieves this by downsampling the input image and fusing the resulting feature maps from various layers. It allows the merging of different details from different levels of spatial detail, enhancing the network's ability to capture comprehensive and contextually rich representations of the input data. By enabling information exchange at multiple spatial scales, the MLSP module contributes to the network's capacity for more robust and effective feature extraction. The MLSP module utilizes distinct branches to aggregate features from various layers, capturing different perceptual fields and information at multiple scales (Fig. 2). In the first step of our proposed MLSP module, we generate two images of different resolutions by employing bilinear interpolation on the input image. These images are then processed by a feature fusion layer with shared weights, extracting features and generating a feature map with a channel dimension of 16 at various scales. This process allows for the construction of a spatial pyramid, incorporating information from different resolutions into the subsequent stages of the network. In the second step, feature maps from various scales in the spatial pyramid are concatenated to achieve multi-scale information fusion. Finally, the feature maps in the constructed multi-level pyramid are enhanced and aggregated using the Mixer Layer, which optimally utilizes them within the BiLSTM module.

### BiLSTM for coarse feature fusion

Integrating the Transformer into 3D reconstruction has led to the inclusion of a global attention mechanism in the feature extraction module of many MVS networks. This mechanism enables improved consideration of global information. However, it also results in increased computational complexity and heightened sensitivity to noise, which can adversely affect the quality of 3D reconstruction. As a solution, we suggest the utilization of BiLSTM in the feature fusion module instead of the Transformer. This approach enables us to prioritize long-range semantic features, increase resistance to interference, improve the capacity for generalization, and ultimately elevate the quality of 3D reconstruction. In the BiLSTM module, we implemented long-range dependencies using LSTM in both vertical and horizontal directions, similar to the Vision Transformer, while also reducing the number of parameters.

#### BiLSTM module

**Figure 3:**
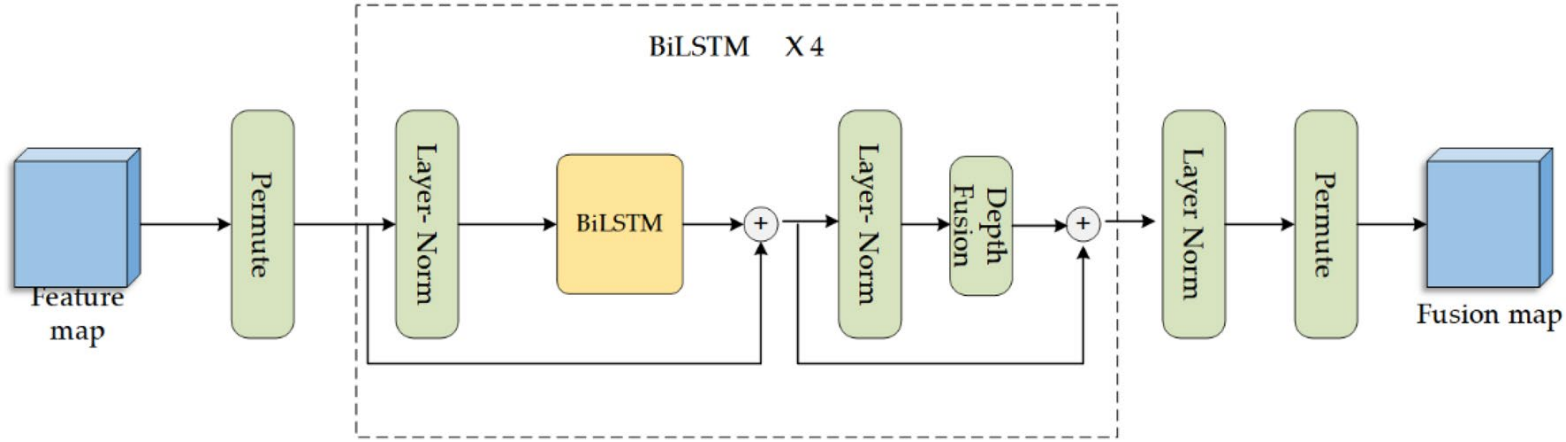
The structure of BiLSTM Module.

We propose to apply BiLSTM to the low-resolution feature map fusion module. The BiLSTM module is mainly composed of the Layer-norm^24^ block, BiLSTM block, Depth Fusion block, and Residual connection, as shown in Fig. 3. For the sake of future reference, we define the input low-resolution feature map as $p_{i} \in {\mathbb{R}}^{C \times H \times W}$. To align with the Layer-norm block used in the Transformer, the input to the BiLSTM block requires restructuring, denoted as $\left\{ {H \times W \times C} \right\}$. Initially, the permute function is employed to modify the channel structure of the input feature map, resulting in the restructured representation denoted as $P \in {\mathbb{R}}^{H \times W \times C}$. Subsequently, the modified feature maps were passed into the BiLSTM block, where two residual joins were executed. Finally, the output is processed by the Layer-norm block, and then the permute function is used to restore it to its original channel structure, which is denoted as $\left\{ {C \times H \times W} \right\}$.

#### BiLSTM block

**Figure 4:**
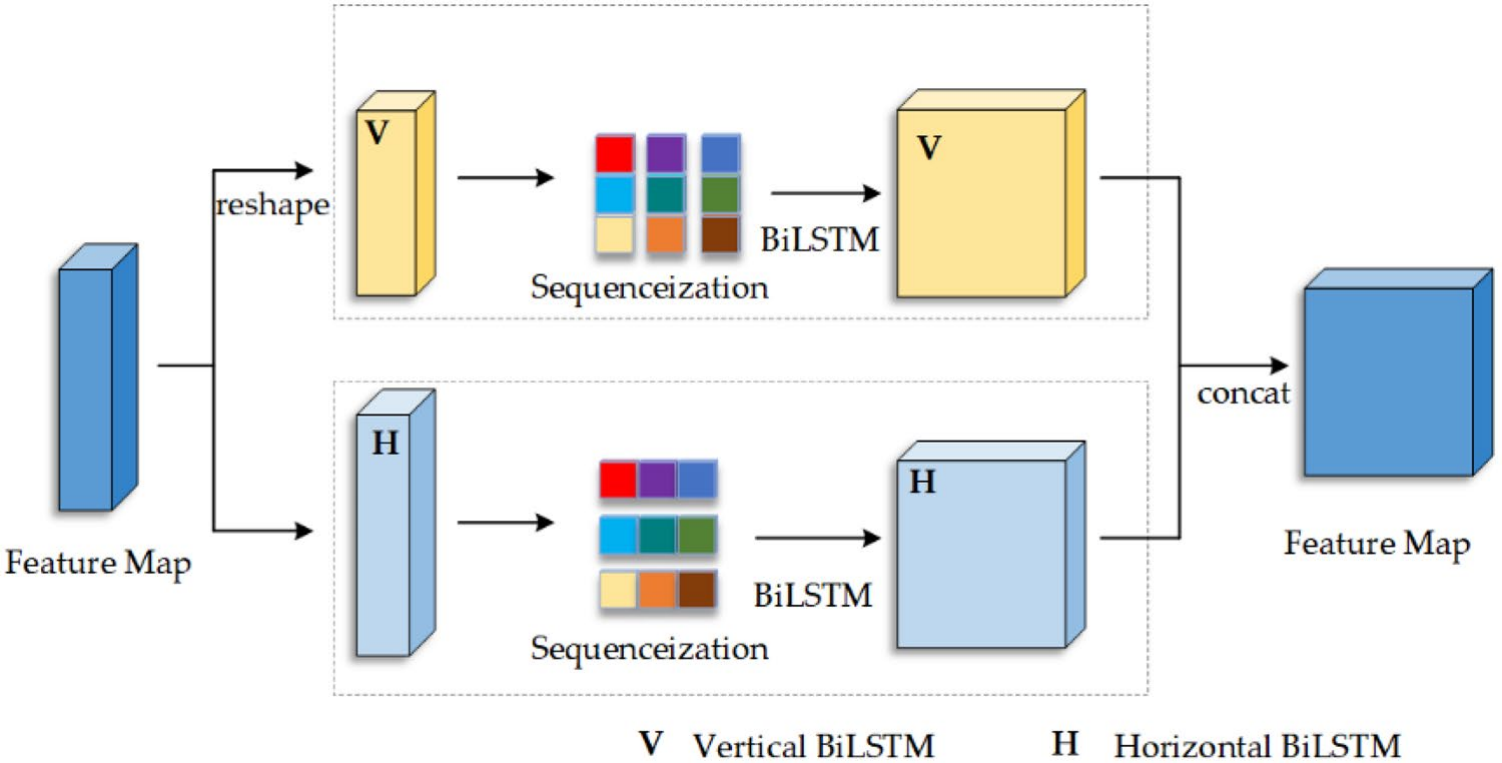
The structure of BiLSTM Block.

The BiLSTM block consists of two separate BiLSTM layers that process feature sequences in the row and column directions, as illustrated in Fig. 4 below. The combination of the two individual LSTMs^25^ forms a BiLSTM layer. LSTMs belong to a distinct category of recurrent neural networks (RNNs) that exhibit proficiency in capturing long-range relationships and mitigating the challenge of vanishing or exploding gradients related to distant connections.

Taking the BiLSTM layer in the vertical direction as an example, if the input sequence of the BiLSTM is defined as $\overrightarrow {H}$, then $\overleftarrow {H}$ is an inverted rearrangement sequence of $\overrightarrow {H}$. The $\overrightarrow {H}$ sequence is input into one of the ordinary LSTMs, and the corresponding LSTM is named Forward LSTM. The sequence, $\overleftarrow {H}$, is input into another standard LSTM, which is commonly known as the Backward LSTM. The Backward LSTM handles the input sequence in reverse order. The resulting output, represented as $\overrightarrow {{Y_{for} }}$, from the Forward LSTM, and the output, denoted as $\overleftarrow {{Y_{back} }}$, from the Backward LSTM, are subsequently concatenated together. Both $\overrightarrow {{Y_{for} }}$ and $\overleftarrow {{Y_{back} }}$ have a channel dimension of D. The final concatenated output, $Y$, has a channel dimension of 2D. This splicing operation combines the information from both the forward and backward directions, enabling the model to capture bidirectional dependencies and leverage them for improved performance. The resulting spliced output, $Y$, with an increased channel dimension, provides richer and more comprehensive information for subsequent stages of the model.

1 $$ \overrightarrow {{Y_{for} }} = LSTM_{forward} (\overrightarrow {H} ) $$

2 $$ \overleftarrow {{Y_{back} }} = LSTM_{backward} (\overleftarrow {H} ) $$

3 $$ Y = concatenate(\overrightarrow {{Y_{for} }} ,\overleftarrow {{Y_{back} )}} $$

In the preliminary step, the input characteristic map is specified as $P \in {\mathbb{R}}^{H \times W \times C}$. In utilizing the BiLSTM network, the characteristic map must be serialized. Serialization involves converting the multidimensional characteristic map into a sequential representation. As shown in Fig. 4 below for the Vertical BiLSTM module and the Horizontal BiLSTM module, the number of tokens in the height and width directions are W and H, respectively.

During the subsequent step, the feature map sequence is separately fed into the BiLSTM block along the width and height directions. Where $\left\{ {P_{:,w,:} \in {\mathbf{\mathbb{R}}}^{H \times C} } \right\}_{w = 1}^{W}$ represents a group of sequences entered horizontally, W represents the aggregate count of sequences entered horizontally, and C represents the number of channels. All input sequences $P_{:,w,:}$ are passed through the weight-sharing Vertical BiLSTM block:

4 $$ Y_{:,w,:}^{ver} = BiLSTM\left( {P_{:,w,:} } \right) $$

Similarly, $\left\{ {P_{h,:,:} \in {\mathbf{\mathbb{R}}}^{W \times C} } \right\}_{h = 1}^{H}$ represents the set of vertically-oriented input sequences, H represents the total count of vertically-oriented input sequences, and C represents the number of channels. These inputs are all fed into the weight-sharing Horizontal BiLSTM block:

5 $$ Y_{h,:,:}^{hor} = BiLSTM\left( {P_{h,:,:} } \right) $$

In the third step, $Y^{ver} \in {\mathbf{\mathbb{R}}}^{H \times W \times 4D}$ and $Y^{hor} \in {\mathbf{\mathbb{R}}}^{H \times W \times 4D}$ are concatenated in the channel dimension, where D represents the hidden dimension of the BiLSTM block. This concatenated feature map is then propagated through a fully connected layer feedforward network. That is, $FC( \bullet )$ in the following equation accomplishes channel fusion, yielding the ultimate output feature map $Y \in {\mathbf{\mathbb{R}}}^{H \times W \times C}$.

6 $$ Y_{hidden} = concatenate\left( {Y^{ver} ,Y^{hor} } \right) $$

7 $$ Y = FC(Y_{hidden} ) $$

#### Depth Fusion

In the BiLSTM block, the input feature map needs to be serialized in horizontal and vertical directions. The resulting sequences are then separately fed into the corresponding BiLSTM layer in each direction. Finally, the outputs from both directions are stitched together. The described process may limit the network's ability to effectively combine local and global features, leading to a potential lack of generalization. To enhance the feature representation further, we propose the utilization of a deep fusion module that combines convolutional operations with a Bottleneck-like approach to fuse the resulting feature maps. This fusion module aims to capture and integrate multi-scale information effectively, leading to improved feature representation capabilities.

**Figure 5:**
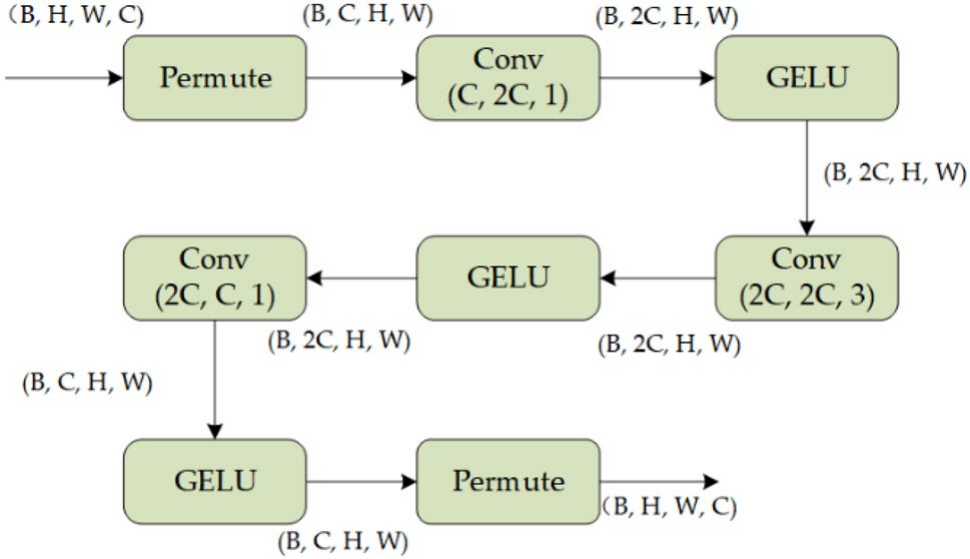
The structure of Depth Fusion Block.

The Depth Fusion Block is visually represented in Fig. 5 below. In the initial phase, a 1 × 1 convolutional kernel is employed to expand the channel dimension of the feature map, doubling its original size while preserving the feature map scale. Subsequently, a convolutional kernel of size three performs the same mapping while preserving the channel dimension and the feature map scale unchanged. A convolution kernel of size one is employed to restore the original channel dimension. The GELU activation function is applied throughout the module.

### Depth inference for MVS

Sampling occurs at the lowest resolution level, perpendicular to the direction normal to the reference viewpoint, assuming widely spaced depths, as set up in CVPMVSNet. To acquire the ultimate depth value of a point, the output probability volume is aggregated along the depth axis through averaging. The probabilities associated with pixel points corresponding to different depths undergo a weighting process based on their respective depth values, followed by aggregation.

8 $$ D^{i} \left( x \right) = \sum\limits_{m = 0}^{M - 1} {dX_{x}^{i} (d)} $$

To improve the accuracy of the depth map, our network leverages the lowest resolution depth map as prior information and applies Bicubic interpolation to upsample the initial rough depth map. Building upon this approach, the depth hypothesis is progressively refined to construct a cost volume pyramid. This pyramid enables continuous optimization of the depth map while adding finer details.

### Loss function

In line with the coarse-to-fine multi-stage MVS method, we employ the L1 loss as a supervised signal. This involves sampling the true depth map into the layer pyramid depth map and calculating the absolute distance between the true and predicted depths. The loss function is calculated as follows:

9 $$ Loss = \sum\limits_{i = 0}^{I - 1} {\sum\limits_{X \in \Omega } {\left\| {{\mathbf{D}}_{GT}^{i} \left( x \right) - {\mathbf{D}}^{i} (x)} \right\|_{i} } } $$

In this context, the notation Ω represents the collection of valid pixel points. At the same time, i signifies the ith level of the pyramid, ${\mathbf{D}}_{GT}^{l} \left( p \right)$ is the actual depth value of pixel x at level i, and ${\mathbf{D}}^{i} (x)$ is the predicted depth value of pixel x at level i.

## Experiments

### Datasets

**Figure 6:**
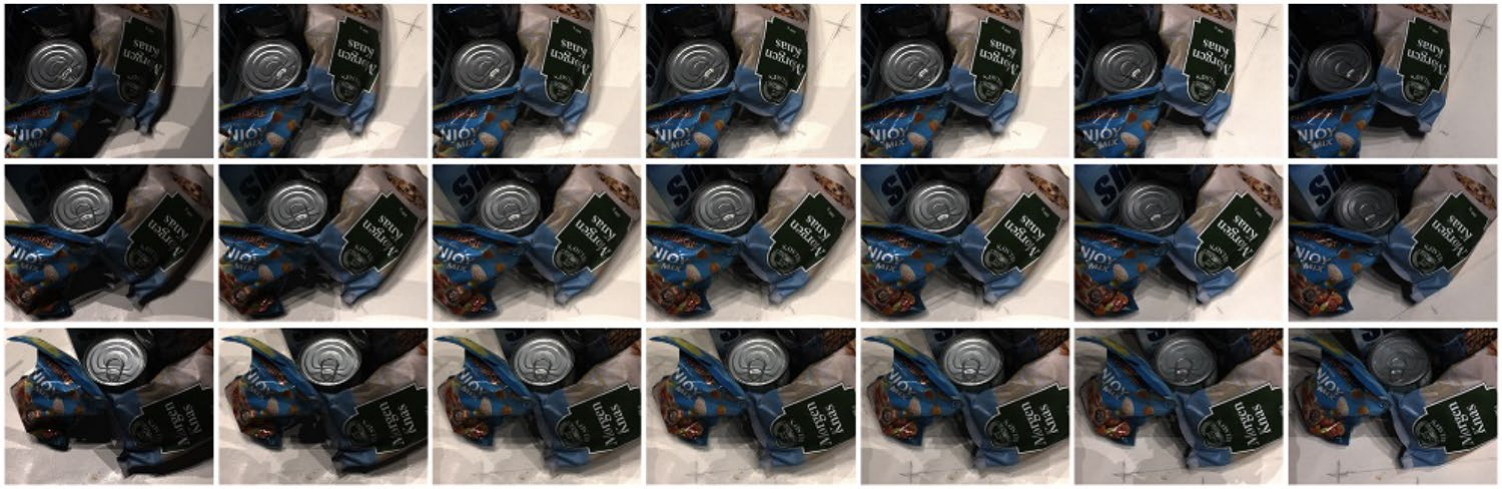
DTU dataset partial visualization.

Our network is trained and tested using the DTU dataset, which is a publicly available dataset. This dataset utilizes an industrial robot arm with adjustable luminance lights to capture photographs of objects from various viewpoints. Each viewpoint in the DTU dataset is precisely controlled, ensuring that a 3D point cloud is acquired using a structured light sensor. This allows for offline evaluation of the point cloud and facilitates easy monitoring of experimental results. The DTU dataset comprises 124 distinct scenes, each captured from either 49 or 64 viewpoints. These viewpoints cover a range of seven different lighting conditions, encompassing various geometries and texture structures in the scenes. As an illustration, Fig. 6 displays the scan96 scenes within the dataset. The images are arranged from left to right, showcasing the images captured at seven distinct luminance levels. Moreover, the images are vertically arranged in sequential order, signifying that they correspond to images taken from unique vantage points.

**Figure 7:**
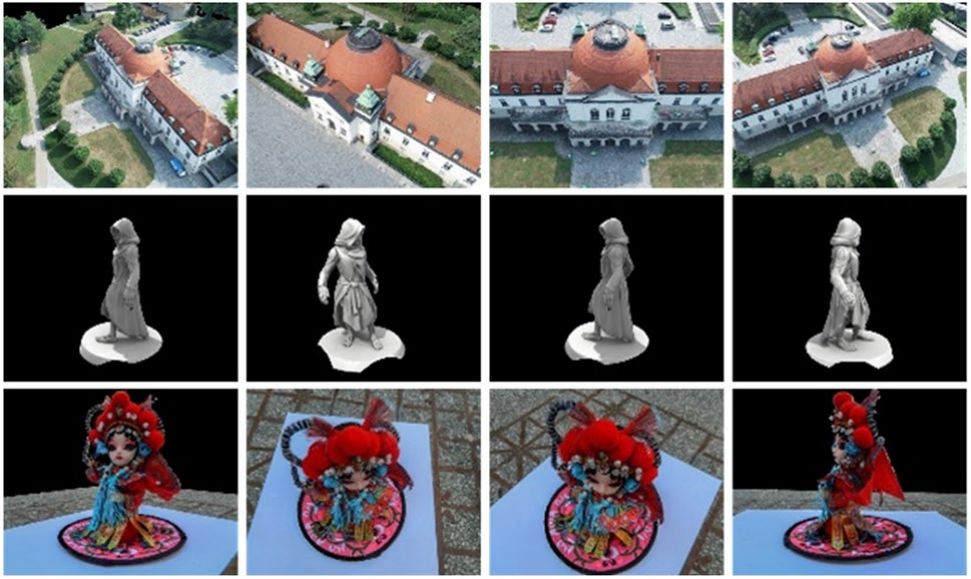
BlendedMVS dataset partial visualization.

As depicted in Fig. 7, presented underneath, the BlendedMVS dataset encompasses a diverse collection of 113 scenes with varying scales, and the number of views ranges from 20 to 1000. In contrast to the DTU dataset, which utilizes a fixed number of views, the BlendedMVS dataset employs multiple cameras to capture random shots. Additionally, depth sensors are used to accurately measure depth information. By incorporating randomness in viewpoint selection and incorporating accurate depth measurements, the BlendedMVS dataset aims to simulate real-world scenarios more effectively, enabling better performance and generalization of algorithms trained on it. However, this dataset does not provide a true value point cloud and does not allow for point cloud evaluation. Therefore, we solely utilize the BlendedMVS dataset for qualitative evaluation and visualization result presentation.

### Metrics

For evaluating the point cloud model generated by our network, three selected metrics are Accuracy (Acc), Completeness (Comp), and Overall score (Overall). These metrics are employed to evaluate the precision, comprehensiveness, and overall fidelity of the reconstructed point cloud in comparison to the ground truth reference. Each metric is measured in millimeters, and decreased values for these metrics correspond to improved algorithm effectiveness. This means that a smaller value for Accuracy (Acc), Completeness (Comp), and Overall score (Overall) indicates a better reconstruction quality of the point cloud generated by the algorithm. The objective is to minimize these metrics, indicating a stronger alignment of the reconstructed point cloud with the ground truth data.

10 $$ Acc = \frac{1}{\left| P \right|}\sum\limits_{x \in P} {\mathop {\min }\limits_{y \in G} } \left\| {x - y} \right\|^{2} $$

11 $$ Comp = \frac{1}{\left| G \right|}\sum\limits_{x \in G} {\mathop {\min }\limits_{y \in p} } \left\| {x - y} \right\|^{2} $$

12 $$ Overall = \frac{Acc + Comp}{2} $$

In this context, P represents the points in the predicted point cloud model, while G represents all the points in the real point cloud.

### Implementation details

The experimental environment consisted of an AMD Ryzen 7 5800X 8-Core Processor as the CPU, 32 G.B. of memory, and an NVIDIA GeForce TITAN RTX GPU with 24 G.B. of video memory. The deep learning framework used was PyTorch, version 1.7.1, with CUDA version 10.1 for GPU acceleration. For training and testing, the image width is 160, height is 128 and the number of input views is 3. For model optimization, the Adam optimizer was employed with hyperparameters set to β1 = 0.9 and β2 = 0.999.

### Experimental performance

#### Results on DTU dataset

Table 1 presents a comprehensive analysis of the algorithm discussed in our model and other learning-based MVS methods using the DTU dataset. The comparison is based on objective metrics, and it provides insights into the performance of our algorithm concerning other existing methods. In terms of runtime and parameter count, our model has achieved satisfactory results, demonstrating relatively low runtime and reasonable parameter count. This balance enhances the practical feasibility and efficiency of our method in real-world applications.

**Table 1:** Comparison of results on DTU Dataset.

Method	Acc. (mm)↓	Comp. (mm)↓	Overall (mm)↓	Params (M)↓	Runtime (s)↓
Colmap^26^	6.5778	10.1405	8.2930	-	-
R-MVSNet^27^	1.0896	1.4115	1.2506	0.80	0.051
TransMVSNet^11^	1.0248	1.3075	1.1662	1.15	0.097
CasMVSNet^15^	1.4045	1.6096	1.5071	0.93	**0.022**
CVP-MVSNet^16^	1.1964	1.0569	1.1267	0.55	0.067
AACVP-MVSNet^12^	1.1329	**0.8874**	1.0071	**0.54**	0.064
GeoMVSNet^17^	1.1406	1.8218	1.4812	15.00	0.695
Ours	**0.9285**	0.9879	**0.9582**	0.72	0.036

Significant values are in bold.

**Figure 8:**
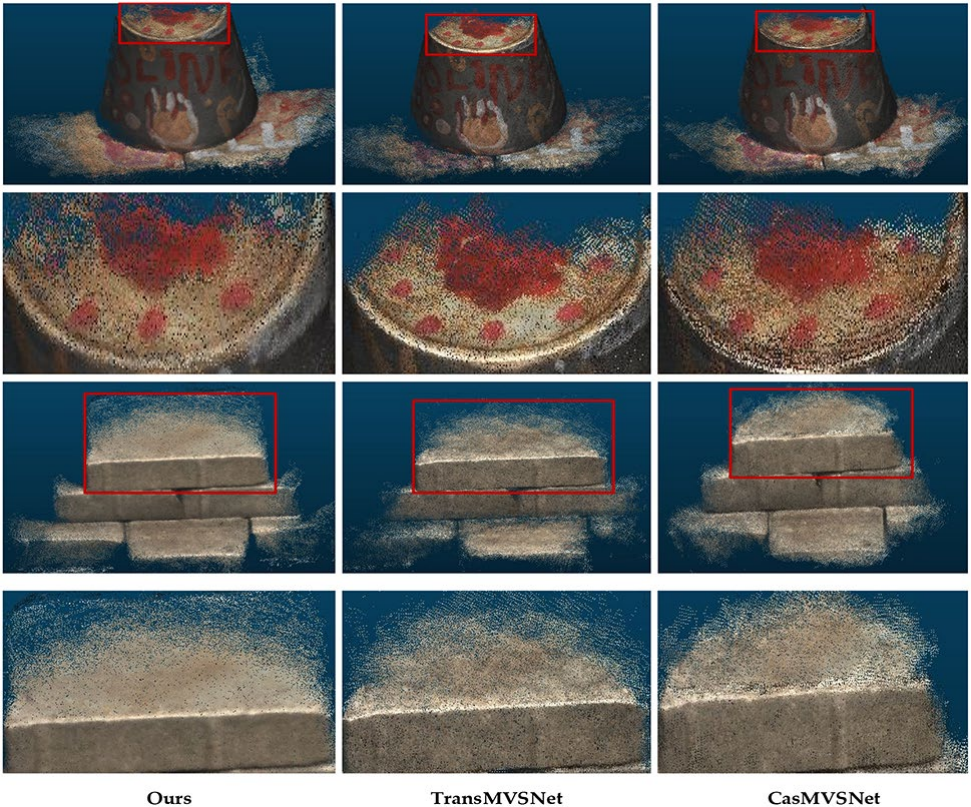
Comparison of results on DTU dataset.

Colmap is a classical MVS algorithm that reconstructs 3D models by iteratively establishing correspondences between image pairs. This step-by-step approach allows Colmap to gradually build the 3D model by leveraging the detected correspondences. In the case of low-resolution images, the Colmap method may encounter challenges in the feature point matching phase, leading to sparse correspondences and, subsequently, poor reconstruction results. R-MVSNet^27^ Replaces 3DCNN with GRU for Recurrent Regularisation. TransMVS is a method that adopts a Transformer-based approach for MVS tasks. Cas-MVSNet, on the other hand, utilizes a cascaded multi-scale cost volume strategy combined with adaptive depth sampling. CVP-MVSNet, in contrast, employs a compact and lightweight network architecture to construct pyramids of cost volumes, enabling higher-resolution 3D reconstruction. AACVP-MVSNet, in contrast, incorporates an attention layer into the network architecture to enhance feature extraction. Due to the extensive computational resources required by GeoMVSNet^17^, we did not retrain the model but conducted direct testing. Regarding accuracy and overall performance, as shown in Fig. 8, the algorithm proposed in this paper showcases superior results compared to other methods.

#### Results on BlendedMVS dataset

**Figure 9:**
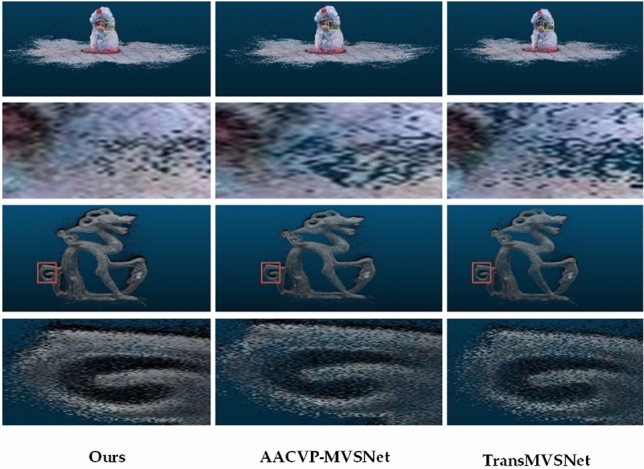
Comparison of results on BlendedMVS dataset.

To comprehensively evaluate the generalization capability of our proposed model, we reconstruct the scene from the BlendedMVS dataset without conducting any fine-tuning on the network explicitly trained on the DTU dataset. The results are shown in Fig. 9 below, where the significantly overall reconstructed point cloud of this network shows greater density in the visualization output when compared to the other networks.

#### Ablation study

1. Effectiveness of Different Components

Our proposed network incorporates the MLSP module, which facilitates the construction of a multi-level spatial pyramid. This design enables the network to interact with image information at different scales, capturing holistic and detailed information. By leveraging the multi-level spatial pyramid, our algorithm can effectively incorporate contextual information and capture the hierarchical structure of the input data. We integrate the BiLSTM module into our model to enable semantic information filtering. This block allows the model to selectively focus on the most relevant and helpful information for the task of 3D reconstruction. By incorporating the BiLSTM module, our model becomes more adept at capturing and utilizing meaningful semantic information, which enhances the quality and accuracy of the generated 3D reconstructions. The effectiveness of all our proposed modules is validated through ablation experiments, and the corresponding results are presented in Table 2. The comprehensive model that incorporates all the proposed modules demonstrates superior performance across all metrics, attaining optimal results.

2. Number of Different BiLSTM Modules

**Table 2:** Quantitative performance with different components.

MLSP	BiLSTM	Acc. (mm)↓	Comp. (mm)↓	Overall (mm)↓
		1.1964	1.0569	1.1267
√		0.9635	1.0257	0.9946
√	√	**0.9285**	**0.9879**	**0.9582**

Significant values are in bold.

To account for the varying requirements of the BiLSTM module, we investigated to assess the impact of selecting different numbers of layers on the results. Based on the observations outlined in Table 3, a discernible pattern emerges whereby the metrics exhibit a decline to a certain degree as the number of layers in the BiLSTM module increases. However, beyond a specific range of layer configurations, the metrics start to increase. Consequently, the best results were achieved when utilizing four layers in the BiLSTM module. This indicates that four layers strike an optimal balance between model complexity and performance, resulting in the most favorable outcomes for the task at hand.

3. Effectiveness of Depth Fusion

**Table 3:** Ablation study on the number of Different BiLSTM modules.

BiLSTM	Acc. (mm)↓	Comp. (mm)↓	Overall (mm)↓
2	0.9563	1.0163	0.9863
4	**0.9285**	**0.9879**	**0.9582**
6	0.9421	0.9912	0.9666

Significant values are in bold.

As the BiLSTM Module utilizes BiLSTM to process feature sequences in the row and column directions, it is necessary to serialize the input feature. Our proposed BiLSTM Module integrates an additional feature fusion block. This block is positioned before the output and aims to fuse and enhance semantic information across different temporal and spatial dimensions. As shown in the data in Table 4, Depth-CNN indicates the use of depth-separable convolution for feature fusion, while Linear indicates the use of linear layers for feature fusion. Based on the experimental results, it is evident that the proposed Depth Fusion block, which incorporates a BottleNeck-like structure, outperforms other methods in all metrics. The block indicates that the chosen architecture and design of the Depth Fusion block are highly effective in achieving superior performance across various evaluation metrics. The BottleNeck-like structure likely contributes to the optimal fusion of depth information, resulting in enhanced results for the task under consideration.

4. Effectiveness of BiLSTM Module

**Table 4:** Ablation study on depth fusion.

Depth fusion	Acc. (mm)↓	Comp. (mm)↓	Overall (mm)↓
Depth-CNN	0.9369	1.0096	0.9733
Linear	0.9342	1.0078	0.9710
Ours	**0.9285**	**0.9879**	**0.9582**

Significant values are in bold.

In our proposed network, we incorporate the BiLSTM module for feature aggregation following the MLSP module. This design allows for effectively integrating and aggregating features from multiple spatial scales. The BiLSTM module plays a crucial role in leveraging contextual information and capturing long-range dependencies among the features, leading to improved performance in tasks that require a comprehensive understanding of the input data. By combining the MLSP and BiLSTM modules, our network benefits from multi-scale feature representation and contextual modeling, enhancing its overall capability for the given task. Table 5 primarily investigates the substitution of the BiLSTM module in our proposed network with either an attention mechanism or a convolutional neural network.

**Table 5:** Results on different modules.

Methods	Acc. (mm)↓	Comp. (mm)↓	Overall (mm)↓
Vanilla-SA^6^	0.9269	1.0120	0.9708
LSDA^28^	0.9732	1.0738	1.0235
SSA^29^	0.9756	1.0400	1.0078
SSA-IWSA^13,29^	**0.9266**	1.0244	0.9755
Linear-SA-soft^30^	0.9295	1.0145	0.9720
Control-SA^31^	0.9557	1.0387	0.9972
ConvNext-Depth^20^	0.9486	1.0139	0.9813
ConvNext-Block^20^	0.9608	1.0191	0.9899
Incep-Block^22^	0.9784	1.0248	1.0016
ConvNext-Incep^20^	0.9894	1.0163	1.0028
Ours	0.9285	**0.9879**	**0.9582**

Significant values are in bold.

In Table 5, Vanilla-SA, LSDA, and SSA represent initial self-attention, long-short distance attention^28^, and scaling attention^29^. In Table 5, SSA-IWS^13^ refers to alternate scaling and interactive window attention, while Linear-SA-soft represents linear attention^30^. Control-SA is to perform feature extraction using a network that freezes pre-trained weights along with self-attention and then fuses the extracted feature maps. ConvNext-Depth applies ConvNext architecture with four layers featuring hidden dimensions that progressively grow. On the other hand, ConvNext-Block denotes the usage of ConvNext with four layers of the exact dimensions. ConvNext is a convolutional implementation of the SwimTransformer and ResNet-like structure. It has performed superior to SwimTransformer and ResNet in various classification and image detection tasks. This indicates that ConvNext improves accuracy and effectiveness in capturing and representng features in the context of these tasks.

**Figure 10:**
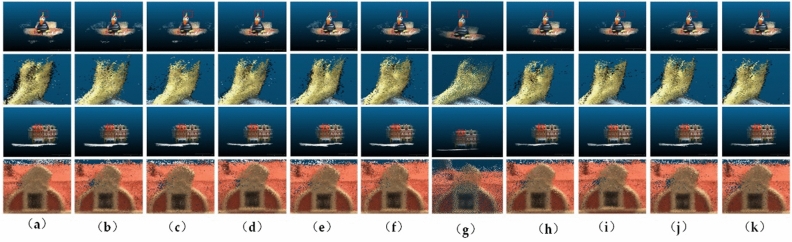
Results on different Modules. (**a**) ours; (**b**) vanilla-SA; (**c**) LSDA; (**d**) SSA; (**e**) SSA-IWSA; (**f**) SSA-IWSA-soft; (**g**) Control-SA; (**h**) ConvNext-depth; (**i**) ConvNext-Block; (**j**) Incep-Block; (**k**) ConvNext-Incep.

Incep-Block denotes a structure that employs InceptionNext as the primary architecture. ConvNext-Incep indicates a structure that alternates between using ConvNext and InceptionNext within the network. The metric results (Fig. 10) indicate that our proposed structure, the BiLSTM module, achieves optimal performance in completeness and objectivity metrics. This suggests that the BiLSTM module effectively captures and incorporates relevant information, resulting in comprehensive and accurate results for the given task.

3. Explore Cost Volume Regularization

We compared these networks' cost volume regularization modules to facilitate a more comprehensive comparison between convolutional neural networks and Vision Transformer-based neural networks. In Table 6, 3Dvit refers to using 3DTransformer, while 3DVit-BN indicates using 3D Transformer with BatchNorm regularization. The experimental metrics demonstrate that convolutional networks (3DCNN) for cost volume regularization achieve optimal performance across all metrics. In Table 6, 3Dvit refers to using 3DTransformer, while 3DVit-BN indicates using 3D Transformer with BatchNorm regularization. The experimental metrics demonstrate that convolutional networks (3DCNN) for cost volume regularization achieve optimal performance across all metrics.

**Table 6:** Results on different regularization modules.

Block	Acc. (mm)↓	Comp. (mm)↓	Overall (mm)↓
3Dvit	0.9538	1.0192	0.9865
3DVit-BN	1.0531	1.0411	1.0471
Ours	**0.9285**	**0.9879**	**0.9582**

Significant values are in bold.

## Conclusion

We introduce a MVS network with Bidirectional Semantic Information explicitly designed for 3D reconstruction on low-resolution images. We propose utilizing an MLSP module during the feature extraction phase to establish a spatial pyramid, enabling the interaction of various spatial information. Additionally, we utilize the BiLSTM module to enable the interaction of feature representations across diverse locations. By comparing the BiLSTM module with different attention mechanisms, it outperforms them in objective metrics and visual representation. This experimental verification of BSI-MVS showcased a significant enhancement in the precision of 3D reconstruction. But our network is primarily designed for low resolution. In the future, we will delve into the research of high-resolution 3D reconstruction networks and hope to further reduce the network parameters while improving the accuracy of 3D reconstruction.

## Data Availability

Our network uses two publicly available datasets, BlendedMVS dataset and DTU dataset. And BlendedMVS dataset is available at https://github.com/YoYo000/BlendedMVS, DTU dataset is available at https://roboimagedata.compute.dtu.dk/?page_id=36.

## References

[1] Liu, J. *et al.* PlaneMVS: 3D Plane Reconstruction from Multi-View Stereo. in *Proceedings of the IEEE Computer Society Conference on Computer Vision and Pattern Recognition* vols 2022-June 8655–8665 (2022).

[2] Hirschmüller H (2008). Stereo processing by semiglobal matching and mutual information. IEEE Trans. Pattern Anal. Mach. Intell..

[3] Yao, Y., Luo, Z., Li, S., Fang, T. & Quan, L. MVSNet: Depth inference for unstructured multi-view stereo. In *Lecture Notes in Computer Science (including subseries Lecture Notes in Artificial Intelligence and Lecture Notes in Bioinformatics)* vol. 11212 LNCS 785–801 (2018).

[4] Wei, Z., Zhu, Q., Min, C., Chen, Y. & Wang, G. AA-RMVSNet: Adaptive aggregation recurrent multi-view stereo network. in *Proceedings of the IEEE International Conference on Computer Vision* 6167–6176 (2021).

[5] Vaswani A (2017). Attention is all you need. Adv. Neural Inf. Process. Syst..

[6] Dosovitskiy, A. *et al.* an Image Is Worth 16X16 Words: Transformers for Image Recognition At Scale. arXiv:2010.11929 (2020).

[7] Schuster M, Paliwal KK (1997). Bidirectional recurrent neural networks. IEEE Trans. Signal Process..

[8] Tatsunami, Y. & Taki, M. Sequencer: Deep LSTM for image classification. arXiv arXiv:2205.01972 (2022).

[9] Kar A, Häne C, Malik J (2017). Large-scale data for multiple-view stereopsis. Adv. Neural Inf. Process. Syst..

[10] Yao, Y. *et al.* BlendedMVS: A large-scale dataset for generalized multi-view stereo networks. In *Proceedings of the IEEE Computer Society Conference on Computer Vision and Pattern Recognition* 1787–1796 (2020).

[11] Ding, Y. *et al.* TransMVSNet: Global context-aware multi-view stereo network with transformers. In *Proceedings of the IEEE Computer Society Conference on Computer Vision and Pattern Recognition* vols 2022-June 8575–8584 (2022).

[12] Yu A (2021). Attention aware cost volume pyramid based multi-view stereo network for 3D reconstruction. ISPRS J. Photogramm. Remote Sens..

[13] Liao, J. *et al.* WT-MVSNet: Window-based transformers for multi-view stereo. arXiv arXiv:2205.14319 (2022).

[14] Zhang, J., Yao, Y. & Quan, L. Learning signed distance field for multi-view surface reconstruction. In *Proceedings of the IEEE International Conference on Computer Vision* 6505–6514 (2021).

[15] Gu, X. *et al.* Cascade cost volume for high-resolution multi-view stereo and stereo matching. In *Proceedings of the IEEE Computer Society Conference on Computer Vision and Pattern Recognition* 2492–2501 (2020).

[16] Yang, J., Mao, W., Alvarez, J. M. & Liu, M. Cost volume pyramid based depth inference for multi-view stereo. In *Proceedings of the IEEE Computer Society Conference on Computer Vision and Pattern Recognition* 4876–4885 (2020).

[17] Zhang, Z., Peng, R., Hu, Y. & Wang, R. GeoMVSNet: Learning multi-view stereo with geometry perception. *Proc. IEEE Comput. Soc. Conf. Comput. Vis. Pattern Recognit.* **2023**-**June**, 21508–21518 (2023).

[18] Lee, J. Y., DeGol, J., Zou, C. & Hoiem, D. PatchMatch-RL: Deep MVS with pixelwise depth, normal, and visibility. In *Proceedings of the IEEE International Conference on Computer Vision* 6138–6147 (2021).

[19] Cao, C., Ren, X. & Fu, Y. MVSFormer: Multi-view stereo by learning robust image features and temperature-based depth. arXiv arXiv:2208.02541 (2022).

[20] Liu, Z. *et al.* A ConvNet for the 2020s. In *Proceedings of the IEEE Computer Society Conference on Computer Vision and Pattern Recognition* vols 2022-June 11966–11976 (2022).

[21] Liu, Z. *et al.* Swin Transformer: Hierarchical vision transformer using shifted windows. In *Proceedings of the IEEE International Conference on Computer Vision* 9992–10002 (2021).

[22] Yu, W., Zhou, P., Yan, S. & Wang, X. InceptionNeXt: When inception meets ConvNeXt. *arXiv* arXiv:2303.16900 (2023).

[23] Wei, Z., Zhu, Q., Min, C., Chen, Y. & Wang, G. Bidirectional hybrid LSTM based recurrent neural network for multi-view stereo. *IEEE Trans. Vis. Comput. Graph.* 1 (2022). 10.1109/TVCG.2022.3165860 35394911

[24] Ba, J. L., Kiros, J. R. & Hinton, G. E. Layer Normalization. arXiv arXiv:1607.06450 (2016).

[25] Shi X (2015). Convolutional LSTM network: A machine learning approach for precipitation nowcasting. Adv. Neural Inf. Process. Syst..

[26] Schonberger, J. L. & Frahm, J. M. Structure-from-motion revisited. In *Proceedings of the IEEE Computer Society Conference on Computer Vision and Pattern Recognition* vols 2016-Decem 4104–4113 (2016).

[27] Yao Y (2019). Recurrent MVSnet for high-resolution multi-view stereo depth inference. Proc. IEEE Comput. Soc. Conf. Comput. Vis. Pattern Recognit..

[28] Wang, W. *et al.* CrossFormer++: A versatile vision transformer hinging on cross-scale attention. arXiv arXiv:2303.06908 (2023). 10.1109/TPAMI.2023.3341806 38113150

[29] Yang, R. *et al.* ScalableViT: Rethinking the context-oriented generalization of vision transformer. In *Lecture Notes in Computer Science (including subseries Lecture Notes in Artificial Intelligence and Lecture Notes in Bioinformatics)* vol. 13684 LNCS 480–496 (2022).

[30] Katharopoulos, A., Vyas, A. & Pappas, N. Transformers are RNNs: Fast autoregressive transformers with linear attention. In *Proceedings of the 37th International Conference on Machine Learning* (2020).

[31] Zhang, L. & Agrawala, M. Adding conditional control to text-to-image diffusion models. arXiv arXiv:2302.05543 (2023).

